# Editorial: Components Separation Techniques in Abdominal Wall Surgery

**DOI:** 10.3389/jaws.2025.15997

**Published:** 2026-01-06

**Authors:** René H. Fortelny, Lars Nannestad Jørgensen

**Affiliations:** 1 General Surgery, Sigmund Freud Private University Vienna, Vienna, Austria; 2 Digestive Disease Center, Bispebjerg Hospital, University of Copenhagen, Copenhagen, Denmark

**Keywords:** abdominal wall reconstruction, complex ventral and incisional hernias, component separation techniques, hernia surgery, open anterior and posterior approaches

Component separation techniques (CST) have become an essential part of abdominal wall reconstruction, providing reliable myofascial advancement for complex ventral and incisional hernias. The primary objective of CST is restoration of the midline and re-establishment of functional core stability. The available CST methods now include open anterior and posterior approaches, minimally invasive and endoscopic variants, hybrid procedures, and combined releases tailored to anatomical characteristics.

Since the first description of the anterior CST by Albanese [1] and its popularization by Ramirez et al. [2], several refinements have been made. Anterior CST enables medialization through external oblique release but requires extensive subcutaneous dissection, resulting in increased wound morbidity. This limitation promoted the transition toward posterior CST, which preserves perforators, minimizes soft-tissue trauma, and allows for retromuscular mesh placement—a strategy associated with improved long-term durability.

Posterior CST was further advanced with the introduction of transversus abdominis release (TAR)by Novitsky et al [3]. TAR facilitates wide lateral release and creation of a continuous retromuscular plane suitable for large meshes. It has become the preferred method for extensive defects, recurrent hernias, and loss-of-domain cases, demonstrating low recurrence rates between depending on complexity [4].

Endoscopic anterior CST represents an important minimally invasive alternative [5]. By preserving perforating vessels and avoiding wide subcutaneous flaps, it reduces wound morbidity compared with the open anterior approach. However, its medialization potential remains more limited, and the learning curve is considerable. Additionally, patient selection is more restricted, as severe scarring, prior lateral releases, or large defects may limit its applicability. Nevertheless, when applied appropriately, endoscopic ACS offers a valuable option within the CST armamentarium.

A further adjunct increasingly used in complex cases is intraoperative fascial traction [6]. Various devices and traction protocols have been developed to promote progressive medialization during surgery, particularly in large or rigid defects. Fascial traction can be combined with posterior CST or TAR to facilitate midline closure, reduce tension, and avoid excessive lateral releases. Early data suggest promising reductions in defect tension, although standardized indications and protocols are still lacking.

Despite the expanding CST toolbox, generating a universal treatment algorithm remains difficult. Differences in defect morphology, tissue quality, prior operative history, patient comorbidities, and surgeon expertise create considerable variability between cases. While attempts have been made to classify defects and match them to specific CST techniques, the heterogeneity of abdominal wall pathology and the rapid evolution of new methods limit the feasibility of a strictly applied algorithm. Instead, individualized treatment planning based on anatomical, functional, and technical considerations remains essential.

Minimally invasive and robotic techniques continue to broaden the reach of CST. Robotic TAR (rTAR) has demonstrated advantages including enhanced visualization, improved ergonomics, and reduced postoperative morbidity [7]. Recurrence outcomes appear comparable to open TAR, although access to robotic platforms and procedural costs remain limiting factors. Structured training pathways are required as these technologies gain prominence.

Optimal outcomes require thorough patient optimization. Obesity, malnutrition, diabetes, smoking, and sarcopenia significantly increase postoperative risk. Prehabilitation—including nutritional support, metabolic control, and physical conditioning—is increasingly recognized as essential. Adjunctive strategies such as botulinum toxin A injections and progressive pneumoperitoneum aid in loss-of-domain scenarios by reducing closure tension and improving abdominal compliance [8].

Mesh selection remains a critical element of CST-based reconstruction. Permanent synthetic mesh used in the retromuscular plane provides durable reinforcement in clean settings, while biologic and biosynthetic meshes may be considered for contaminated or high-risk fields. TAR’s ability to create a large, vascularized retromuscular space promotes excellent mesh integration and long-term stability.

Challenges persist, including variability in surgical technique and inconsistency in terminology, which complicate comparison across published studies. Functional outcomes—such as abdominal wall strength, core stability, and health-related quality of life—remain underreported relative to recurrence. Standardized reporting frameworks and multicenter registries will be essential to refine indications and compare CST techniques.

Future developments in CST will likely benefit from advanced imaging, quantitative CT-based reconstruction planning, artificial intelligence–assisted prediction models, and biomaterials such as patient-specific 3D-printed meshes. Integration of these innovations into clinical practice must be guided by robust long-term evidence.

In summary, CST has matured into a versatile reconstructive strategy for complex abdominal wall defects. Posterior CST and TAR remain the cornerstone of modern reconstruction, while minimally invasive and endoscopic techniques offer alternative approaches in selected cases. Adjuncts such as intraoperative fascial traction further expand the reconstructive armamentarium. Continued innovation, improved standardization, and emphasis on functional outcomes will be essential for further advancement of the field.
